# Membrane Vesicles of *Pectobacterium* as an Effective Protein Secretion System

**DOI:** 10.3390/ijms222212574

**Published:** 2021-11-22

**Authors:** Joanna Jonca, Malgorzata Waleron, Paulina Czaplewska, Aleksandra Bogucka, Aleksandra Steć, Szymon Dziomba, Jacek Jasiecki, Michał Rychłowski, Krzysztof Waleron

**Affiliations:** 1 Laboratory of Plant Protection and Biotechnology, Intercollegiate Faculty of Biotechnology UG and MUG, University of Gdansk, Abrahama 58, 80-307 Gdansk, Poland; joanna.jonca@ug.edu.pl; 2 Laboratory of Mass Spectrometry-Core Facility Laboratories, Intercollegiate Faculty of Biotechnology UG and MUG, University of Gdansk, Abrahama 58, 80-307 Gdansk, Poland; paulina.czaplewska@ug.edu.pl (P.C.); lewad.ola@gmail.com (A.B.); 3 Department of Toxicology, Faculty of Pharmacy, Medical University of Gdansk, 107 Hallera Street, 80-416 Gdansk, Poland; aleksandra.stec@gumed.edu.pl (A.S.); szymon.dziomba@gumed.edu.pl (S.D.); 4 Department of Pharmaceutical Microbiology, Faculty of Pharmacy, Medical University of Gdansk, Al. Gen. Hallera 107, 80-416 Gdansk, Poland; jacek.jasiecki@gumed.edu.pl; 5 Laboratory of Virus Molecular Biology, Intercollegiate Faculty of Biotechnology UG and MUG, University of Gdansk, Abrahama 58, 80-307 Gdansk, Poland; michal.rychlowski@biotech.ug.edu.pl

**Keywords:** bacterial membrane vesicles, *Pectobacterium*, proteomic analysis, plant pathogens, bacterial virulence

## Abstract

Bacteria of genus *Pectobacterium* are Gram-negative rods of the family *Pectobacteriaceae*. They are the causative agent of soft rot diseases of crops and ornamental plants. However, their virulence mechanisms are not yet fully elucidated. Membrane vesicles (MVs) are universally released by bacteria and are believed to play an important role in the pathogenicity and survival of bacteria in the environment. Our study investigates the role of MVs in the virulence of *Pectobacterium*. The results indicate that the morphology and MVs production depend on growth medium composition. In polygalacturonic acid (PGA) supplemented media, *Pectobacterium* produces large MVs (100–300 nm) and small vesicles below 100 nm. Proteomic analyses revealed the presence of pectate degrading enzymes in the MVs. The pectate plate test and enzymatic assay proved that those enzymes are active and able to degrade pectates. What is more, the pathogenicity test indicated that the MVs derived from *Pectobacterium* were able to induce maceration of *Zantedeschia* sp. leaves. We also show that the MVs of β-lactamase producing strains were able to suppress ampicillin activity and permit the growth of susceptible bacteria. Those findings indicate that the MVs of *Pectobacterium* play an important role in host-pathogen interactions and niche competition with other bacteria. Our research also sheds some light on the mechanism of MVs production. We demonstrate that the MVs production in *Pectobacterium* strains, which overexpress a green fluorescence protein (GFP), is higher than in wild-type strains. Moreover, proteomic analysis revealed that the GFP was present in the MVs. Therefore, it is possible that protein sequestration into MVs might not be strictly limited to periplasmic proteins. Our research highlights the importance of MVs production as a mechanism of cargo delivery in *Pectobacterium* and an effective secretion system.

## 1. Introduction

Bacteria of genus *Pectobacterium* are Gram-negative, rod-shaped facultative anaerobes with peritrichous flagella [1,2]. Earlier grouped into the *Enterobacteriaceae* family, they recently have been reclassified to the family *Pectobacteriaceae* within the Enterobacteriales order [3]. They are phytopathogens of broad host range specificity that are able to cause economically significant soft rot and blackleg diseases in plants [4]. They infect both mono-and dicotyledonous plant hosts, which include important crops such as chicory, banana, potatoes, carrots, sugar beets, sunflowers, and ornamental plants belonging to families of *Araceae*, *Asteraceae*, *Begoniaceae* and *Cactaceae* [5]. They were also isolated from soil, water samples and gastrointestinal tracts of invertebrates [6]. Their ability to macerate plant tissue and cause disease is governed by the presence of several plant cell wall degrading enzymes (PCWDEs) that are secreted mainly through a type II secretion system (T2SS) [7,8]. Their expression is regulated via a quorum sensing (QS) mechanism [9]. The most important PCWDEs are pectinases. These enzymes are responsible for the induction of soft rot symptoms due to degradation of the middle lamella and primary plant cell walls [2]. Pectins are one of the main constituents of a plant cell wall [10]. They are a family of complex polysaccharides that contain α-1,4 galacturonic acid residues [11]. The most abundant in plants is polygalacturonate (PG), a linear chain of α-1,4-linked D-galacturonate residues, methyl-esterified in about 70–80% [10,12]. Pectinases cleave the glycosidic bonds or ester bonds of the pectic polymers, thus depolymerizing the pectic chain. The enzymes that act on PG involve polygalacturonases, pectin lyases and pectate lyases with either endo- or exo-cleaving activity [10].

Membrane vesicles (MVs) are spherical particles of 20–250 nm diameter on average and consist of a hydrosol enclosed by one or two biological membranes [13]. They can be divided into outer membrane vesicles (OMVs) and outer-inner membrane vesicles (O-IMVs), depending on their formation mechanism. Outer membrane vesicles (OMVs) are formed by bulging of the bacterial outer membrane and encapsulating the content of the periplasmic space. As such, they consist of outer cell wall constituents, including lipoproteins, phospholipids, and lipopolysaccharide (LPS) [14]. Recently, it was also found that bacteria can produce O-IMVs that are formed through protrusion of both the cytoplasmic and outer bacterial membranes and also contain the cytoplasmic components of a bacterial cell [15]. O-IMVs were first discovered in *Shewanella vesiculosa* [16,17].

Although initially believed to be a consequence of membrane instability, membrane vesicles (MVs) release is now recognized as a novel bacterial secretion system [18]. MVs are produced in various environments, including water environments, biofilms, and in a range of hosts, including humans [14,19]. This secretory pathway provides multiple advantages, such as the protection of proteins from degradation due to encapsulation in lipid vesicles, the possibility of simultaneous release of several different effector proteins at a target location, and enhanced delivery into recipient cells through a membrane fusion [20].

The mechanism of OMVs and O-IMVs formation has yet not been fully understood. It is suggested that MVs form as a consequence of an imbalance between membrane tension and osmotic pressure that leads to an extracellular protrusion of the membrane areas where peptidoglycan is detached [13]. The imbalance may be due to a disrupted lipid asymmetry [21]. Mutants lacking or with limited expression of OmpA, Tol-Pal complex proteins, known to link the outer membrane to the peptidoglycan layer, or altered number of Brown’s lipoprotein (Lpp)-peptidoglycan (PG) crosslinks, have a hypervesiculation phenotype [19,22,23]. MVs may also be formed due to the accumulation of peptidoglycan fragments and misfolded proteins [15,18,21].

Until recently, the research on MVs has been concentrated mainly on the study of the MVs produced by human pathogenic bacteria and their role in disease [19]. It was found that MVs may deliver proteases and toxins to limit the growth of other competing bacteria, and DNA material that can transform the recipient cells. Moreover, they mediate cellular adhesion and biofilm formation. They are also able to deliver bacterial virulence factors and toxins into host cells and tissues, modulating the immune response of the host, and are able to aid in its evasion [24].

The production of MVs was discovered in several plant pathogens [25,26,27]. Studies have also revealed that MVs of plant pathogens carry many microbe-associated molecular patterns (MAMPs), similarly to human pathogenic bacteria [28]. They include lipopolysaccharide (LPS), peptidoglycan (PG), flagellin and elongation factor Tu (EF-Tu) [25,26]. MVs were capable of inducing the immune response in *Arabidopsis thaliana*, dependent on the brassinosteroid-insensitive 1–associated kinase (BAK1) and the suppressor of BAK1-interacting receptor-like kinase 1 (SOBIR1) coreceptors [20]. MVs also seem to be the alternate delivery route for extracellular enzymes excreted through T2SS and possibly type III (T3SS) secretion systems [25,26,28]. Recently, the protein content of *Pectobacterium* MVs has been described for *P. betavasculorum* [29] and *P. brasiliense* [30]. The analyses revealed a presence of several proteins involved in a host-pathogen interplay, including proteases, pectate lyases and oligoglycan transporter (KdgM).

In this study, we investigated broader the role of MVs in *Pectobacterium* pathogenesis. For that purpose, we isolated membrane vesicles from three *Pectobacterium* strains, *P. zantedeschiae*, *P. odoriferum*, and *P. versatile*. Further, we characterised the MVs by transmission electron microscopy (TEM), SDS-PAGE and proteomic analyses. We assessed the production of MVs, depending on the cultivation media. We also investigated how a GFP protein overexpression affects the production of MVs, using GFP-tagged strains. We showed that pectin degrading enzymes, an important virulence factor present in MVs, retain their biological activity. What is more, MVs are able to induce soft rot disease symptoms in *Zantedeschia* sp. (Calla lily) leaves. We also showed that the MVs derived from a strain resistant to ampicillin, when added to a growth medium, can protect bacteria susceptible to ampicillin from the growth inhibitory effects exerted by this antibiotic. Our work provides a new insight into the cargo content of *Pectobacterium* MVs, and their importance in the virulence of these bacteria. What is more, our study also sheds light on the mechanism of MVs formation and the sequestration of cargo into membrane vesicles.

## 2. Results

### 2.1. MVs Characterization by Electron Microscopy

**Figure 1:**
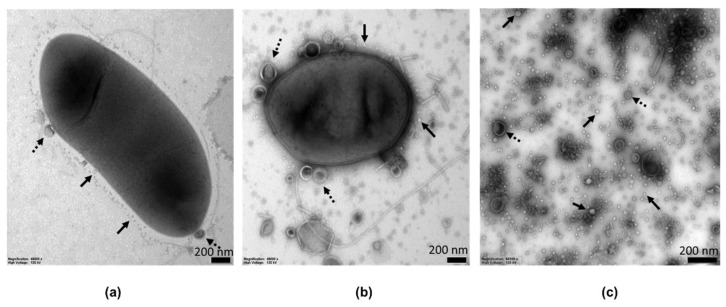
Membrane vesicles (MVs) from *P. zantedeschiae* 9M culture. TEM of bacteria grown on (**a**) the MH agar supplemented with 10% sucrose and (**b**) the CVP agar. Solid arrows indicate small MVs of 10–30 nm size budding off from the cells. Dashed arrows indicate large vesicles of about 50-100 nm in diameter. The size bar is 200 nm. (**c**) TEM images of purified MVs derived from a planktonic culture of *P. zantedeschiae* 9M grown in the M63 medium supplemented with 0.2% glycerol and 0.4% PGA. Solid arrows indicate two distinct subpopulations of MVs of sizes 10–30 nm and 100–300. Dashed arrows indicate vesicles with a double membrane, similar to O-IMVs. The size bar is 200 nm.

In our studies of the role of MVs in *Pectobacterium*, we used three different *Pectobacterium* strains, *P. zantedeschiae* 9M, *P. odoriferum* Car 1 and *P. versatile* DPMP180. All studied strains produced MVs. In addition, *P. zantedeschiae* 9M strain grown on two different agar media was analysed by transmission electron microscopy (TEM). The obtained micrographs (Figure 1a,b and Figure S1) show membrane vesicles budding off from the cells. The vesicles differ in morphology, depending on the growth medium. Bacteria cultivated on MH agar plates supplemented with 10% sucrose produced mainly small vesicles of about 10–30 nm size, packed with electron-dense material. Only a few large vesicles of 50–100 nm size were visible in the images. Bacteria grown on CVP agar plates also produced small vesicles of 10–30 nm size, and additionally many large vesicles of sizes ranging from 100 to 300 nm. Vesicles enclosed with two membranes, similar to outer-inner membrane vesicles (O-IMVs), were also visible in the micrographs.

To better visualise the vesicles, they were isolated from planktonic bacterial cultures. For that purpose, bacteria were grown in a liquid M63 medium supplemented with 0.2% glycerol and 0.4% PGA. This medium is similar in composition to the CVP agar, and it has the advantage of being a defined mineral medium. To minimise the contamination of MVs with cell debris due to cell lysis, the MVs were harvested in the late logarithmic growth phase. The analysis of TEM images revealed similar vesicles to those seen when bacteria were grown on the CVP agar (Figure 1c and Figure S2). Two main subpopulations of MVs were present, large vesicles of about 100–300 nm size and small spherical MVs that carried electron-dense material of sizes ranging from 10 to 30 nm. Some large vesicles possessed a double membrane. The contamination of MVs preparations by particles resembling fragments of flagella could also be seen in some images.

### 2.2. The Comparison of MVs Production in Different Cultivation Media

In order to compare the effect of the medium composition on MVs production, *P. zantedeschiae* 9M strain was grown in six different media. The lipid content of MVs was assessed by the DPH method, normalised to the log CFU mL^−1^ and expressed as relative to the lipid content of vesicles harvested from M63 medium supplemented with 0.2% glycerol and 0.4% PGA.

**Figure 2:**
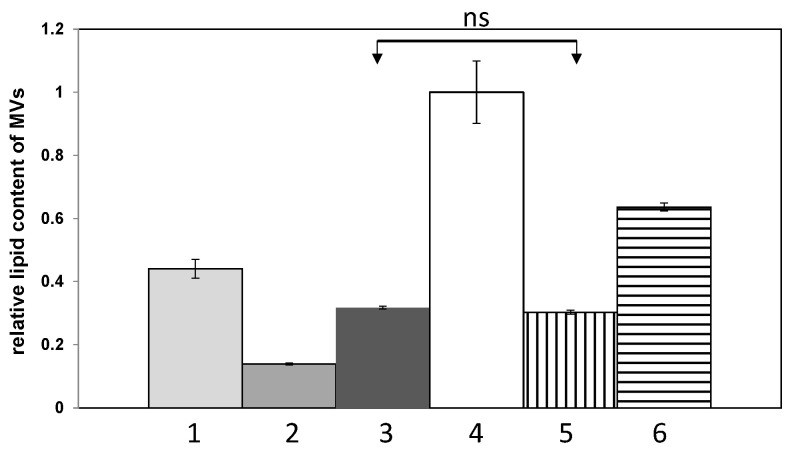
The comparison of MVs production, expressed as the overall amount of released lipids, depending on the medium composition. MVs were purified from *P. zantedeschiae* 9M strain grown in six different media: 1-M63 with 0.2% glycerol and 10% chicory extract, 2-M63 with 0.2% glycerol and 1% sucrose, 3-M63 with 0.2% glycerol, 4-M63 with 0.2% glycerol and 0.4% PGA, 5-M63 with 0.2% glycerol and 10% Calla lily extract, 6-M63 with 0.2% glycerol and 10% potato extract. The lipid content of MVs was assessed by the DPH method, normalized to the log CFU mL^−1^ and expressed as relative to the lipid content of vesicles harvested from the M63 medium supplemented with 0.2% glycerol and 0.4% PGA. One way ANOVA and Tukey’s test were used for analyses. The data represent one of two separate, reproducible experiments presented as mean ± standard deviation of the mean (SEM). The abbreviation ‘ns’ means not significant.

The production of MVs, expressed as the overall lipid content released in the vesicles, depended strongly on the medium composition (Figure 2). MVs production was the highest in the PGA supplemented medium. On the other hand, MVs production in the M63 medium containing only 0.2% glycerol without PGA was about 3.2 (±0.3) times lower than in the M63 medium supplemented with 0.2% glycerol and 0.4% PGA. It indicates that PGA is a strong inductor of the vesicle formation. The lowest MVs production was in the M63 medium supplemented with 1% sucrose, about 7.6 (±0.5) times less than the MVs production in the PGA supplemented medium.

### 2.3. The MVs Production by Wild-Type and GFP-Tagged Pectobacterium Strains

**Figure 3:**
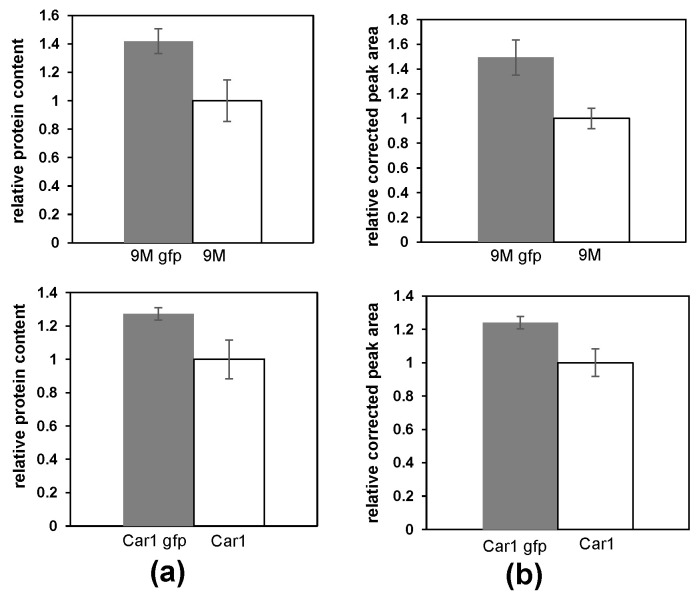
The comparison of the MVs production by *P. zantedeschiae* 9M gfp (top) and *P. odoriferum* Car1 gfp (bottom) relative to the wild-type strains. (**a**) The protein content of the samples was assessed by the BCA method. (**b**) Corrected peak areas were calculated from the CE analysis. The obtained results were expressed as relative to the MVs derived from the wild-type strains. The data represent one of two (Car1) or three (9M) separate reproducible experiments, expressed as mean ± SEM. Statistical significance was calculated using Student’s *t* test (*p* < 0.05).

One of the proposed mechanisms of vesicles formation is a budding of the membrane in the areas where misfolded proteins or protein cargo accumulate [19]. GFP-tagged *Pectobacterium* strains constitutively overexpress the GFP protein. It was therefore hypothesised that they might produce more MVs or their MVs might carry more proteins. In order to test this hypothesis, the production of MVs by the GFP-tagged *P. zantedeschiae* 9M and *P. odoriferum* Car1 strains was compared with the MVs production from the wild-type strains. The comparison was performed by assessing the protein content of the MVs, and with capillary electrophoresis (CE). The CE measurements allowed to assess the overall increase in the number or size of the vesicles. Additionally, the lipid content of the MVs from *P. odoriferum* Car1 strain was assessed with the DPH method. All methods indicated that the MVs production was markedly increased in the GFP-tagged strains (Figure 3 and Figure S3). The observed differences were statistically significant. The protein content of the MVs was on average 1.4 times higher (*p* = 0.0075), while the corrected peak area was 1.5 times higher (*p* = 0.0003) for *P. zantedeschiae* 9M gfp in comparison to the wild-type strain. The protein content of the MVs was 1.3 times higher (*p* = 0.0029), while the corrected peak area was on average 1.2 times higher (*p* = 0.0007) for *P. odoriferum* Car1 gfp MVs compared to the wild-type strain. The lipid content of the MVs was 1.3 times higher (*p* = 0.024) for *P. odoriferum* Car1 gfp in comparison to the wild-type strain. The results consistently prove that the presence of the pPROBE-AT-gfp plasmid significantly increased the MVs production for GFP-tagged *Pectobacterium* strains.

### 2.4. The Characterization of MVs from a GFP-Tagged Strain

**Figure 4:**
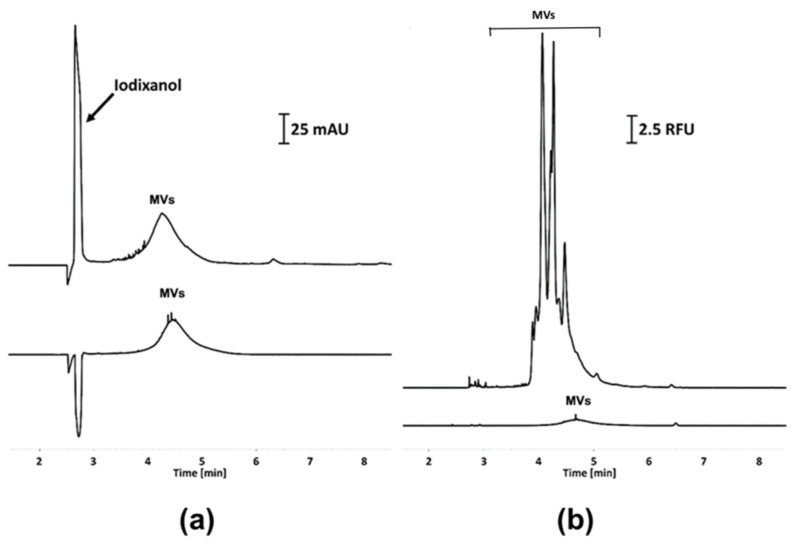
The CE analysis of the MVs isolated from culturing media of (lower traces) the wild-type *P. zantedeschiae* 9M and (upper traces) the GFP-tagged strain. The analyses were performed using (**a**) UV and (**b**) LIF detection. The abbreviations used are: RFU—relative fluorescence unit; AU—absorbance unit.

The analysis of the MVs derived from the GFP-tagged *P. zantedeschiae* 9M strain under the confocal microscope revealed that the strain produced fluorescent MVs (Figure S4). A similar conclusion was drawn from the electrophoretic analysis (Figure 4a,b). The CE-UV of the MVs samples of the wild-type and the GFP-tagged *P. zantedeschiae* 9M strain showed various content of MVs in the samples (Figure 4), which has already been discussed in the Section 2.3 of the manuscript. In turn, the application of LIF detection showed a tremendous difference in detector response in these two types of samples (Figure 4b). The signals registered for the MVs isolated from the wild-type strain, using both detection modes, was considered to be similar in terms of separation efficiency and asymmetry (lower traces in Figure 4). The MVs secreted by the GFP-tagged strain featured significantly stronger fluorescence than the wild-type strain (Figure 4b).

Several signals co-migrating with the main peak were observed in the MVs samples from the GFP-tagged strain (Figure 4b). Similar co-migrating signals were not observed with UV detection (Figure 4a). Only low intense ‘spiky’ signals were registered in the latter case. In our previous works, these signals were assigned to the presence of macromolecular aggregates that are a result of MVs squeezing during ultracentrifugation [29,31]. Thus, it can be concluded that the concentration of fluorescent compounds, co-migrating with the main signal (Figure 4b), is relatively low in relation to MVs, and their detection is a result of significantly greater sensitivity of LIF detector. Their appearance can be attributed to the presence of MVs impurities formed during MVs isolation. It should be stressed that the application of iodixanol cushion provided significant improvement in the isolates’ quality in comparison to the direct ultracentrifugation method. However, utilization of highly sensitive LIF detection was able to detect trace levels of contaminants.

**Figure 5:**
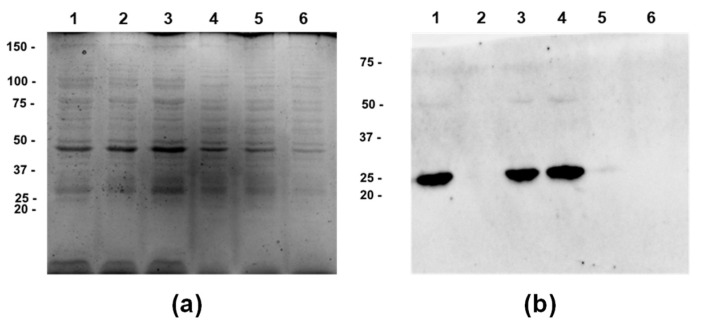
The characterization of the MVs from the GFP-tagged *P. zantedeschiae* 9M strain. (**a**) SDS-PAGE analysis of the MVs harvested from three independent cultures of the wild-type *P. zantedeschiae* 9M (bands 1–3) and the GFP-tagged strain (bands 4–6). Proteins were visualised by a Coomassie Brilliant Blue staining. The GFP protein size is about 27 kDa. Size standards are indicated on the left side of the image (kDa). (**b**) Western blot analysis with anti-GFP monoclonal antibodies of the GFP-tagged *P. zantedeschiae* 9M strain (bands 1, 3, 4) and the wild-type strain (bands 2, 5, 6). Size standards are indicated on the left side of the image (kDa).

The SDS-PAGE analysis of the MVs isolated from three independent cultures of the wild-type *P. zantedeschiae* 9M, and the GFP-tagged strains did not reveal marked differences in bands profile (Figure 5). In both cases, the most abundant were proteins of sizes 30 and 37 kDa. The mass of the GFP protein is about 27 kDa, but the band was not readily discernible on the gel. The western blot analysis, using the anti-GFP monoclonal antibody, revealed a band of about 25 kDa size. Thus, it confirms the presence of the GFP in the MVs from *P. zantedeschiae* 9M gfp. A faint band of about 50 kDa was also visible on the membrane, which most possibly corresponds to a GFP dimer.

### 2.5. Proteomic Analysis

The proteomic analysis of MVs by MALDI-TOF/TOF-MS was conducted for the *P. zantedeschiae* 9M strain grown in two different media, the M63 medium supplemented with 0.2% glycerol and 0.4% PGA, and the M63 medium supplemented with 0.2% glycerol and 10% potato extract. The media for growth were chosen based on the MVs production. *P. zantedeschiae* 9M MVs production, expressed as a total lipid content of MVs, was the highest in those growth media. The proteomic analysis was also conducted for *P. zantedeschiae* 9M gfp that was grown in the M63 medium supplemented with 0.2% glycerol and 0.4% PGA, to assess if the protein composition of MVs would differ after the introduction of the pPROBE-AT-gfp plasmid.

**Figure 6:**
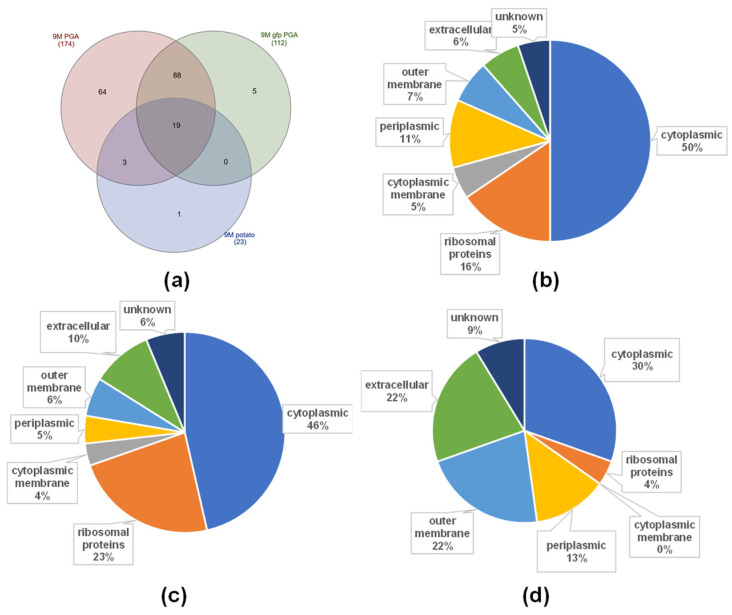
Proteomic analysis of the MVs derived from *P. zantedeschiae* 9M and the GFP-tagged strain cultured in the M63 medium supplemented with 0.2% glycerol and 0.4% PGA, and *P. zantedeschiae* 9M cultured in the M63 medium supplemented with 0.2% glycerol and 10% potato extract. (**a**) Venn diagram of proteins overlapping and exclusive for the three MVs samples. Pie charts depict the percentage of proteins in each localization for the MVs of (**b**) *P. zantedeschiae* 9M, (**c**) the GFP-tagged strains cultured in the M63 medium supplemented with 0.2% glycerol and 0.4% PGA, (**d**) *P. zantedeschiae* 9M cultured in the M63 medium supplemented with 0.2% glycerol and 10% potato extract.

The analysis allowed to identify 172 proteins in the MVs of the wild-type strain, 112 proteins in the MVs of the GFP-tagged strain, and markedly less, 23 proteins in case of the MVs isolated from bacteria grown in the medium supplemented with a potato extract (Figure 6a and Table S1). The cellular localisation of the proteins was determined by the pSORTb software. The majority of detected proteins in the MVs of the wild-type and the GFP-tagged strains grown in the PGA supplemented medium was cytoplasmic (including ribosomal proteins), and comprised 66% and 69% of total proteins detected, respectively (Figure 6b,c). In contrast, the MVs produced by bacteria grown in the medium supplemented with a potato extract carried markedly fewer cytoplasmic proteins. They comprised 34% of the total detected proteins (Figure 6d). Some proteins were present in all three samples, including typical markers of OMVs, such as outer membrane proteins (OMPs) and major outer membrane lipoprotein (Lpp). A Tol-Pal system protein TolB, associated with the membrane stability [23], was also detected. In addition, some important virulence factors were present, such as components of flagella and ABC transporters. What is more, endo-polygalacturonase (PehA), which is a pectate degrading enzyme, was detected in all of the analysed samples. Many ribosomal proteins were also identified. No major differences in the protein composition of the MVs from the wild-type and the GFP-tagged strain were found, 107 proteins overlapped between the MVs from both strains (Figure 6a). Several enzymes that have a role in nutrient acquisition and metabolism were detected in both samples. Moreover, vesicles produced by the GFP-tagged strain contained the GFP protein. A β-lactamase that was also encoded on the plasmid was detected in the analysis. However, it was present beyond the >95% confidence level, most possibly because bacteria were grown without the presence of ampicillin, so its expression was not induced.

### 2.6. Pectate Degrading Activity of MVs

**Figure 7:**
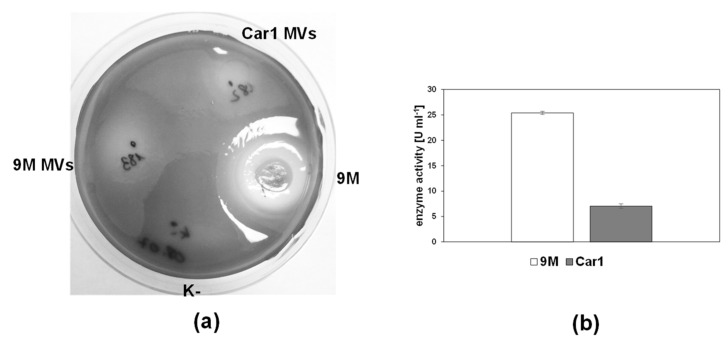
Assessment of pectinase degrading enzymes activity in the MVs from *P. zantedeschiae* 9M and *P. odoriferum* Car1. (**a**) The pectinase plate test on the M63 medium supplemented with 0.4% PGA. *P. zantedeschiae* 9M strain was used as a positive control. Clear halo zones indicate pectate degradation. (**b**) The DNS assay of the MVs samples. The assay measures the presence of reducing sugars. One unit (U) is equivalent to one μmol of product released per min. Bars indicate a standard deviation of the mean, *n* = 3, *p* < 0.05.

Due to the importance of pectate degrading enzymes in *Pectobacterium* virulence, one of the main methods of *Pectobacterium* spp. identification is by confirmation of their ability to degrade pectate on CVP agar plates [32]. In order to assess if the pectate degrading enzymes detected in MVs by proteomic analysis retained their activity, the pectinase plate test was carried out for *P. zantedeschiae* 9M and *P. odoriferum* Car1 strains. The results show that a clear halo zone of degraded pectate was formed where the vesicles were present (Figure 7a). The halo zone was markedly bigger for the MVs of *P. zantedeschiae* 9M than the MVs of *P. odoriferum* Car1. Similar results were obtained in the case of the CVP medium (Figure S6). Cavities were deep in case of the MVs of *P. zantedeschiae* 9M, whereas the MVs of *P. odoriferum* Car1 strain produced large, but shallow cavities.

In order to measure the activity of pectate enzymes, the DNS assay was carried out. The measured activity was 25.4 ± 0.3 U mL^−1^ for MVs of *P. zantedeschiae* 9M, and was significantly higher than the measured activity for MVs of *P. odoriferum* Car1 (7.05 ± 0.4 U mL^−1^) (Figure 7b). These results are consistent with pectinase plate tests and prove that MVs of *Pectobacterium* strains contain different amounts of pectate degrading enzymes.

### 2.7. MVs Pectate Degrading Activity on Zantedeschia sp. Leaves

The ability of MVs to macerate plant tissues without the presence of bacteria was assessed with the plant pathogenicity test on *Zantedeschia* sp. (Calla lily) leaves. The results show a maceration zone on leaves that were inoculated with the vesicles of *P. zantedeschiae* 9M and *P. odoriferum* Car 1 strains (Figure S6). The MVs of *P. zantedeschiae* 9M were able to induce soft rot disease symptoms after 24 h of incubation. The maceration was less pronounced for the MVs of *P. odoriferum* Car 1 and became visible after 48 h of incubation. These results were consistent with the 3,5-dinitrosalicylic acid (DNS) assay, which indicated that the MVs from *P. odoriferum* Car1 have a lower pectate degrading activity than the MVs of *P. zantedeschiae* 9M. No pectinolytic bacteria were detected in the tissue of the macerated leaves. It indicates that MVs were responsible for the induction of the disease symptoms.

### 2.8. MIC Assay in the Presence of MVs

The presence of β-lactamase enzymes in MVs could imply that they play an active role in bacterial fitness and survival. The MIC assay was carried out to assess if the concentration of β-lactamase in MVs would be significant enough to allow the growth of susceptible bacteria. For that purpose, the MVs derived from two β-lactamase producing *Pectobacterium* strains were added to *Escherichia coli* DH5α grown in the presence of increasing ampicillin concentrations. The GFP-tagged *P. odoriferum* Car 1 strain contained a β-lactamase gene encoded on the pPROBE-AT-gfp plasmid. *P. versatile* DPMP190 strain has a natural resistance to ampicillin. Results of the experiment revealed a significant increase of the MICs of ampicillin for *E. coli* DH5α that was grown in the presence of the MVs in both cases (Figure S7). The growth of this bacterium was detected in up to 600 times higher antibiotic concentrations than without the MVs added. It indicates that β-lactamase producing *Pectobacterium* spp. pack the enzyme into MVs, and those MVs can significantly suppress the antibiotic activity and permit the growth of susceptible bacteria.

## 3. Discussion

*Pectobacterium* genus is one of the ten most important plant pathogens [9]. It causes severe economic losses on agricultural, horticultural and ornamental plants during vegetation, transport, and storage [33]. No effective method of control of this pathogen spread, and treatment for infected plants are yet known [2]. The knowledge of how *Pectobacterium* bacteria interact with their host plants and cause disease would be instrumental in finding new methods to combat bacteria of this genus. However, their virulence determinants were not yet fully elucidated. MVs production in *Pectobacterium* was first reported in 1992 [34]. First characterisations of the MVs of *Pectobacterium* were published in 2020 and 2021 [29,30]. To date, the MVs production was reported for *P. atrosepticum* [34], *P. betavasculorum* [29], and *P. brasiliense* [30]. In our work, we characterise MVs of three other *Pectobacterium* species: *P. zantedeschiae*, *P. ordoriferum* and *P. versatile*. As different species of the genus *Pectobacterium* produce vesicles, MVs can be considered as a universal protein secretion system for these phytopathogens. We also shed additional light on the roles MVs might play in *Pectobacterium* virulence.

MVs were isolated from bacterial cultures in the late logarithmic phase of growth using the ultracentrifugation method with a 40% iodixanol cushion to prevent aggregation of the vesicles, which may occur when MVs are pelleted at the bottom of the centrifugation tube [35]. To remove iodixanol, which would interfere with the analysis by the capillary electrophoresis, MVs samples were subjected to ultrafiltration. It also allowed to eliminate protein contaminants smaller than 300 kDa. However, this crude isolation technique may not prevent contamination with fragments of flagella, which are often pelleted in the ultracentrifugation step [36]. These contaminants were visible in some TEM micrographs.

It is known that yield and cargo of MVs depend on the choice of a growth medium [20,26]. In our investigations, we tried to emulate conditions bacteria encounter when they infect plant tissues. Therefore, we used growth media supplemented with sucrose, plant extracts or PGA. We were able to observe the MVs production on solid media by TEM. The results indicate that the morphology of MVs depends on the medium composition. The population of small MVs (10–30 nm), consisting mainly of OMVs, carried electron-dense material. In addition, large vesicles of 100–300 nm size could be observed. They appeared more abundant in the micrographs of bacteria grown on the medium supplemented with PGA. However, the analysis has its limitations, as it was performed based only on ten TEM images of different fields of view.

The comparison of the MVs production in different growth media was performed with the DPH method. This method was chosen due to its simplicity, high sensitivity, and because it required low amounts of samples. Moreover, it gave comparable results to the CE and BCA methods. The main limitation of all those methods is that if vesicles size distribution varies between samples, it does not provide any information on the relative number of vesicles produced. Nonetheless, it gives a good estimation on the overall lipid expenditure during the vesicle formation or the amount of protein cargo carried in the vesicles. The lipid content of MVs significantly varied, depending on the growth medium. What is worth to note, the lipid content of MVs was the highest in media supplemented with PGA or a potato extract, implicating the role of PGA in the induction of MVs formation. Based on these results and the analysis of TEM images, it can be tentatively postulated that the vesicles of different sizes also differ in regard to their respective cargo. Unfortunately, we were unable to perform proteomic analysis of the MVs of *P. zantedeschiae* grown on sucrose, chicory, and Calla lily supplemented media for comparison due to insufficient MVs production in these media.

The proteomic analyses were conducted for *P. zantedeschiae* 9M and the GFP-tagged strain derived MVs. The number of detected proteins differed in relation to the medium used for bacterial growth. In the case of the potato extract supplemented medium, significantly fewer proteins were detected in the MVs than the PGA supplemented medium.

Our results were similar to those previously reported for *Pectobacterium*, where crude MVs were obtained with different isolation methods and different growth media [29,30]. Several outer membrane markers were found, including Omp proteins, the major outer membrane lipoprotein Lpp, and elongation factors. A Tol-Pal system protein TolB was also present. The Tol-Pal system plays an important role in membrane integrity and is implicated in MVs formation [23]. Microbe associated molecular patterns (MAMPs) that can be recognised by plant pattern recognition receptors (PRRs) were also detected and include peptidoglycan-associated protein (pal), flagellin and elongation factor Tu (Tu-EF) [28]. In addition, many cytoplasmic proteins were found, including GroEL protein, which may indicate OMVs contamination by non-vesicular protein aggregates [36]. On the other hand, a recent discovery of O-IMVs sheds new light on the possible reason for continuous detection of cytoplasmic proteins in membrane vesicles, even despite following rigorous isolation procedures [16,17]. Many of the observed large (100–300 nm) vesicles in the TEM images were similar to O-IMVs. O-IMVs were also observed in [29,30], which may suggest that they are commonly produced by bacteria.

The proteome analysis revealed the presence of several virulence factors, including ABC transporters, oligogalacturonate-specific porin KdgM, and the CdiA toxin, which is a part of the contact-dependent growth inhibition system and plays an important role in bacterial competition [37]. Notably, pectin degrading enzymes, endo-polygalacturonase (PehA) and oligalacturonate lyase (Ogl) were also present. Similarly, to [29,30], the proteomic analysis also revealed the presence of pectate lyases (Pel), but below the 95% confidence level.

The pectinase plate test and DNS assay results proved that pectin degrading enzymes were biologically active, so they were not merely misfolded proteins. What is more, the plant pathogenicity test performed on *Zantedeschia* sp. leaves indicated that the MVs initiated the soft rot disease symptoms. It should be noted that no pectinolytic bacteria were found in the macerated tissues of *Zantedeschia* sp. leaves. Moreover, earlier studies showed that *Pectobacterium* MVs could also cause maceration of potato tubers [30]. Those findings highlight that MVs may play an important role in pathogenicity.

The production of β-lactamases is one of the mechanisms of ampicillin resistance. These enzymes are present in the periplasmic space. Several studies indicated that they might be sequestered into MVs, and exert their protective activity on susceptible bacteria [38,39,40]. We performed an experiment that proved the presence of β-lactam degrading enzymes in the MVs of the ampicillin resistant strains of *Pectobacterium*. The β-lactamase was sequestered into MVs of the GFP-tagged *P. odoriferum* Car1 that contained a β-lactamase gene on the pPROBE-AT-gfp plasmid as well as the MVs of *P. versatile* strain, which has a natural resistance to ampicillin. The MVs of both strains contained a sufficient amount of the enzyme to degrade the antibiotic in the cultivation medium and permit the growth of the ampicillin susceptible *E. coli*. We postulate that it may be an important mechanism of *Pectobacterium* success in various habitats as some β-lactamases are able to degrade lactone rings [41,42], and N-acyl homoserine lactones (AHLs) are signalling molecules in the quorum sensing mechanism. The quorum sensing mechanism plays an essential role in *Pectobacterium* virulence and controls the expression of PCWDEs [8,9]. The expression of quorum quenching molecules is a survival strategy against other bacteria that employ quorum sensing [41].

One of the proposed mechanisms of MVs production is by budding of the bacterial membrane in places where protein cargo accumulates and exerts turgor stress on the membrane [18,19]. Therefore, in strains overexpressing proteins, the yields of MVs should be higher. Moreover, it should lead to the sequestration of those proteins into membrane vesicles, which was proven in the case of periplasmic proteins [18]. In order to check if the MVs production of *Pectobacterium* is affected when bacteria are overexpressing the GFP protein, which should be mainly present in the cytoplasm, MVs production was assessed in the wild-type and the GFP-tagged strains (9M and Car1). For both studied strains, the MVs production was increased when expressing the GFP. Moreover, the presence of the GFP in the MVs was confirmed by the western blot analysis, the proteomic analysis, and the capillary electrophoresis. While recent studies showed that specially engineered GFP proteins may be sequestered into MVs [43], we prove that a native GFP protein may also be delivered into MVs in significant quantities.

To conclude, MVs are a universal secretion system of bacteria of *Pectobacterium* genus. Our experiments prove that *Pectobacterium* species produce OMVs, as well as O-IMVs. Moreover, membrane vesicles are an important factor in their virulence and fitness and are an alternative secretion pathway for the pectate degrading enzymes and a native β-lactamase. What is more, bacteria are also able to pack into MVs cytoplasmic proteins that are not native to the cell, such as a GFP protein and a plasmid originating β-lactamase. Further studies are necessary to assess if different vesicle types also differ in regard to the carried cargo. It would allow to elucidate better the mechanism of MVs formation.

## 4. Materials and Methods

### 4.1. Materials

All materials were purchased from Pol-Aura (Dywity, Poland) unless otherwise stated, and were of analytical grade. Polygalacturonic acid sodium salt (PGA, from citrus fruit, ≥75%), 1,6-diphenyl-1,3,5-hexatriene (DPH, 98%), D-galacturonic acid (monohydrate), ampicillin sodium salt, and diethyldithiocarbamic acid diethylammonium salt (DIECA, 97%) were purchased from Sigma-Aldrich (Saint Louis, MI, USA). Egg yolk lecithin was obtained from Thermo Fisher Scientific (Waltham, MA, USA). Iodixanol (Visipaque) was purchased from GE Healthcare (Chicago, IL, USA). Lysogeny Agar (LA), Muller-Hinton (MH) broth, and phosphate-buffered saline (PBS) were obtained from Graso Biotech (Owidz, Poland). M63 medium was prepared as described in [44]. Crystal Violet Pectate (CVP) medium was prepared according to [32]. The materials for sodium dodecyl sulfate polyacrylamide gel electrophoresis (SDS-PAGE), western blot, and anti-mouse-HRP conjugated antibody were obtained from Bio Rad (Hercules, CA, USA). The anti-gfp antibody (ab 1218) was purchased from Abcam (Cambridge, UK).

### 4.2. Bacteria

*Pectobacterium zantedeschiae* 9M (PCM2893 = DSM105717 = IFB9009), a Type Strain of a new species described by our team, and isolated from *Zantedeschia* sp. [45], was obtained from a collection of the Department of Biotechnology of the Intercollegiate Faculty of Biotechnology University of Gdansk and Medical University of Gdansk. *P. odoriferum* Car1, *P. versatile* DPMP190, and *Escherichia coli* DH5α were obtained from the Department of Pharmaceutical Microbiology Medical University of Gdansk collection of strains. GFP-tagged strains, *P. zantedeschiae* 9M gfp and *P. odoriferum* Car1 gfp were generated by introducing into cells of wild-type strains the pPROBE-AT-gfp plasmid [46,47]. The transformation of bacteria was conducted by electroporation, following a standard protocol for *E. coli* [48].

Bacteria were stored in frozen glycerol stocks at −80 °C and maintained on CVP agar in case of *Pectobacterium* strains without plasmid, or CVP agar with the addition of 200 mg L^−1^ ampicillin if strains carried the pPROBE-AT-gfp plasmid. *E. coli* DH5α was maintained on LA plates.

### 4.3. Preparation of Plant Extracts

Thoroughly washed plants were finely cut or chopped using a mechanical blender, and soaked in an equal quantity of M63 medium supplemented with 0.4% DIECA. Plant debris was removed by filtration and subsequent centrifugation at 85,000× *g* for 0.5 h at 4 °C in a Beckman ultracentrifuge (L7-55, rotor SW28). The extract was then sterilised by filtration through a 0.22 μm nitrocellulose filter (Millipore, Burlington, MA, USA) and stored at −80 °C for further use.

### 4.4. Bacteria Cultivation for Membrane Vesicles Isolation

Bacteria were grown overnight on LA plates at 28 °C. For the cultivation of *P. zantedeschiae* 9M gfp, *P. odoriferum* Car1 gfp, and *P. versatile* DPMP190 strains, the LA medium was supplemented with 200 mg L^−1^ ampicillin. Bacterial suspensions of 0.5 McF were prepared in 0.9% PBS, diluted 300-fold in 200 mL of a growth medium, and incubated at 28 °C with shaking (100 rpm) until the optical density (OD) was about 0.8. The CFU mL^−1^ of bacterial cultures was determined by a standard plate count method.

The type of growth medium used for cultivation was dependent on the experiment. The following growth media were used to determine the MVs production: M63 medium supplemented with 0.2% glycerol, M63 medium supplemented with 0.2% glycerol and 0.4% PGA, M63 medium supplemented with 0.2% glycerol and 10% plant extract. The extracts were prepared from *Cichorium* sp. (Chicory) leaves, *Zantedeschia* sp. (Calla lily), and bulbs of *Solanum tuberosum* (Potato) as described in Section 4.3. For other experiments, bacteria were grown in the M63 medium supplemented with 0.2% glycerol and 0.4% PGA.

### 4.5. Membrane Vesicles Isolation

Bacteria for MVs isolation were grown in broth culture as described in Section 4.4. Bacteria were removed by two subsequent centrifugation steps at 8000× *g* and 10,000× *g*, each for 10 min at 10 °C. The supernatant was collected and passed through a 0.45 μm pore size nitrocellulose filter (Millipore, Burlington, MA, USA). The sterility of the filtrate was assessed by a standard plate count method. The filtrates were concentrated in a Vivaspin 20 (PES membrane, 300 kDa molecular weight cut off (MWCO)) centrifugal concentration system (Sartorius, Göttingen, Germany) at 3000× *g* for 2 h at 10 °C. The concentrated supernatants were diluted with 20 mM Tris-HCl pH 7.4 and layered on the top of a 5 mL cushion consisting of 40% (*v*/*v*) iodixanol. The samples were centrifuged at 85,000× *g* for 4 h at 10 °C in a Beckman ultracentrifuge (L7-55, rotor SW28). The layer of MVs was collected from the top of the iodixanol cushion. The samples were diluted 10-fold, and ultrafiltered in the Vivaspin 20 (PES, MWCO 300 kDa) centrifugal concentrators at 3000× *g* for 3 h at 10 °C, with one washing step to remove residual iodixanol. MVs were diluted in Tris-HCl buffer (pH 7.4) to the final volume of 400 μL and stored at −20 °C.

### 4.6. Microscopy Analyses of MVs

*P. zantedeschiae* 9M strain grown on agar media as well as MVs samples of *P. zantedeschiae* 9M wild-type and GFP-tagged strains were analysed with transmission electron microscopy (TEM). Bacteria were grown on the MH agar supplemented with 10% sucrose or the CVP agar plates for 48 h at 28 °C. MVs were obtained from bacteria grown in the M63 medium supplemented with 0.2% glycerol and 0.4% PGA with the method described in this manuscript (Section 4.5). TEM imaging was performed according to the procedure described by [27]. Briefly, 5 µL of the samples were deposed on the formvar support on a copper mesh (200 mesh, Agar Scientific, Stansted, UK). After solvent evaporation, the sample was contrasted with a 1% uranyl acetate and left for drying. The preparation was investigated with the use of the Tecnai G2 T12 Spirit BioTwin microscope (FEI Company, Hillsboro, OR, USA).

The MVs samples of *P. zantedeschiae* 9M wild-type and GFP-tagged strains were also analysed by confocal fluorescence microscopy. Vesicles were placed on a glass slide, sealed with a coverslip, and examined using the confocal laser scanning microscope (Leica SP8X, Germany) with a 100× oil immersion lens.

### 4.7. Protein Assay

The protein content of MVs samples was quantified by the bicinchoninic-acid assay (BCA) as described in [31]. In brief, the isolates were mixed with 6% SDS solution in a 9 to 1 volume ratio and incubated with BCA kit reagents according to the microplate protocol recommended by the vendor (ThermoFisher Scientific, Waltham, MA, USA). The protein concentration was determined using a calibration curve constructed with bovine serum albumin (BSA) solutions in a range of 0.1–2.0 mg mL^−1^. The measurements were performed with a microplate reader InfiniteM200Pro (Tecan, Männedorf, Switzerland).

### 4.8. Capillary Electrophoresis

Capillary electrophoresis (CE) was performed in uncoated fused silica capillaries (50 µm × 30.2 cm), thermostated at 25 °C. The background electrolyte (BGE) was composed of 50 mM BTP and 75 mM Gly (pH 9.5). During the analysis, 10 kV were applied in positive mode. Samples were injected hydrodynamically for 5 s (3.45 kPa). The UV detection was conducted at 200 and 230 nm for quantitation and peak identity confirmation, respectively. A corrected peak area was used to compare the MVs content of the samples as described in [29]. Fluorescence was measured with a laser-induced fluorescence detector (LIF) using 488 nm laser for excitation and 520 nm filter for emission. A detailed description of the capillary conditioning procedures was described in [31].

### 4.9. DPH Method

The lipid content of MVs samples was assessed by the diphenyl-1,3,5-hexatriene (DPH) assay [49]. DPH was dissolved in dimethylformamide (DMF) to form a stock solution of 2 g L^−1^. The working solution of 0.2 g L^−1^ DPH in H_2_O was prepared immediately prior to the experiment. 50 µL of MVs suspension, 40 µL of 20 mM Tris-HCl buffer solution (pH 7.4), and 10 µL of 200 µg ml^−1^ DPH in H_2_O were added to the 96-well black microplate. Samples were excited at λ = 350 nm, and the fluorescence intensity was monitored at λ = 428 nm in a microplate reader InfiniteM200Pro (Tecan). A standard calibration curve was prepared, using egg yolk lecithin at concentrations of 0, 0.75, 1.5, 3, 6, and 12 g L^−1^.

### 4.10. SDS-PAGE Electrophoresis and Western Blot

MVs samples of the wild-type *P. zantedeschiae* 9M and the GFP-tagged strains were analysed by sodium dodecyl sulfate polyacrylamide gel electrophoresis (SDS-PAGE) on 12% polyacrylamide gel [48]. Samples were prepared in a loading buffer with β-mercaptoethanol, boiled for 5 min in a water bath, and loaded on the gel. After electrophoresis, the gel was stained with Coomassie Brilliant Blue R-250. The presence of the GFP protein was additionally confirmed by the western-blot, using the polyvinylidene difluoride (PVDF) membrane, with anti-GFP antibodies (1:5000 dilution) [48]. The membrane was incubated overnight with the anti-GFP antibodies at 4 °C.

### 4.11. Proteomic Analysis

Proteomic analysis was performed on the isolated MVs samples of the wild-type and the GFP-tagged strains of *P. zantedeschiae* 9M according to the multi-enzyme digestion filter assisted sample preparation (MED-FASP) protocol [50]. The strains were grown in the M63 medium supplemented with 0.2% glycerol and 0.4% PGA. *P. zantedeschiae* 9M strain was additionally grown in the M63 medium supplemented with 0.2% glycerol and 10% potato extract. In each case, the MVs were isolated from three independent cultures and then pooled into one sample for the analysis. The sample was treated with lysate buffer containing 0.1 M Tris-HCl, pH 8.0, 0.05 M DTT, and the lysate was mixed and incubated at 95°C for 10 min. Then, after cooling, due to the low protein concentration, it was completely transferred to a 10 kDa membrane (Microcon, Millipore, Burlington, MA, USA) and treated according to the MED-FASP protocol. Digestion was carried out in the sequence of enzymes: LysC, trypsin, and chymotrypsin. Prior to MS/MS analysis, the sample was purified using StageTips C18 according to the protocol described in [51], and concentrated in a SpeedVac concentrator to a volume of 30 mL. LC-ESI MS/MS analysis was performed on a TripleTOF 5600+ with a DuoSpray Ion Source (Sciex, Framingham, MA, USA) mass spectrometer equipped with an Ekspert MicroLC 200 Plus System (Eksigent, Redwood City, CA, USA). All chromatographic separations were performed on the ChromXP C18CL column (3 µm, 120 Å, 150 × 0.3 mm), and the injection was 5 µL. The chromatographic gradient for each MS run was 11–42.5% B (solvent A 99.9% aqueous solution 0.1% formic acid, solvent B 99.9% acetonitrile 0.1% formic acid) in 60 min. The analysis was controlled by the Analyst software (Analyst TF 1.7.1). MS analysis was conducted in data-dependent acquisition (DDA) mode with precursor ion scan in the range 400–1200 *m*/*z*, followed by fragment ion scans in the range 100–1800 *m*/*z*. Database search was performed with MaxQuant 1.6.2.6a software [52] against the Uniprot *Pectobacterium* database (version from 25.01.21) concatenated with the sequence of GFP protein from *Aequorea victoria*. The specific parameters were: 1% FDR on PSM and protein level, enzymes specified for each sample, cysteine carbamidomethylation as a fixed modification, and methionine oxidation and protein N-terminus acetylation as variable modifications. The mass spectrometry proteomics data have been deposited to the ProteomeXchange Consortium via the PRIDE [53] partner repository with the dataset identifier PXD029090. Proteins were considered significant when a score level was greater than 90, and the number of matched peptides was more than 3. Subcellular locations of the proteins were determined using a PSORTb v3.0.2 software (https://www.psort.org, accessed on 11 September 2021). Venn diagram was created using the InteractiVenn web-based software (www.interactivenn.net, accessed on 11 September 2021) [54].

### 4.12. DNS Assay

Polygalacturonic acid (PGA) solution of 5 g L^−1^ was prepared in 20 mM Tris-HCl buffer solution (pH 7.4). The polygalacturonase activity was determined from the increase in reducing sugars concentration after incubation of the MVs samples from *P. zantedeschiae* 9M and *P. odoriferum* Car 1 strains with the substrate. Reducing sugars content was measured using a modified version of the 3,5-dinitrosalicylic acid (DNS) assay [55]. To perform the assay, 10 μL of MVs samples and 90 μL of 6 g L^−1^ PGA were added to 2 mL test tubes. All tubes were sealed with Parafilm and incubated in a water bath at 30 °C for 30 min. The enzyme reaction was terminated by the addition of 300 μL of DNS solution that consisted of 10 g L^−1^ 3,5-dinitrosalicylic acid and 16 g L^−1^ sodium hydroxide (NaOH). To account for a possible presence of reducing sugars in MVs preparations, blanks were prepared by the addition of MVs to the PGA-DNS mixture. All samples were boiled for 10 min, cooled to room temperature, and diluted 5-fold. The absorbance values were measured at 540 nm in a microplate reader InfiniteM200Pro (Tecan). A calibration curve of D-galacturonic acid standard solutions was prepared at concentrations ranging from 0 to 5 g L^−1^. The final activity was calculated based on the release rate of a galacturonic acid-equivalent reducing sugar from the PGA substrate. One unit (U) is equivalent to one μmol of product released per min.

### 4.13. Pectinase Plate Test

The activity of pectate degrading enzymes in the MVs samples of *P. zantedeschiae* 9M and *P. odoriferum* Car 1 strains was assessed by the pectinase test [56]. The samples were diluted to the same MVs concentration, based on the measurements of corrected peak areas with CE. 30 µL of MVs were dripped on the M63 agar supplemented with 0.4 g L^−1^ PGA. *P. zantedeschiae* 9M strain was used as a positive control. 30 µL of 0.5 McF suspension of bacterial cells from the overnight culture in the LB medium was dripped on the plate. The negative control was 0.9% NaCl solution. After 72 h incubation at 28 °C, the plates were stained by flooding with a 10 % (*w*/*v*) copper acetate. A blue complex is formed with PGA, leaving clear halo zones where the polymer is degraded. The activity of pectate degrading enzymes in MVs was additionally assessed by dripping 30 µL of the MVs of *P. zantedeschiae* 9M and *P. odoriferum* Car 1 strains on the CVP agar plates. The plates were incubated for 72 h at 28 °C. After that time, the formation of cavities due to pectin degradation was assessed visually. The experiments were performed in duplicate.

### 4.14. Plant Pathogenicity Test

Pathogenicity tests were conducted on leaves from healthy plants of *Zantedeschia* sp. (Calla lily). Wounded lesions of about 1 cm length were made with a pipette tip. The lesions were inoculated with 30 µL of MVs derived from *P. zantedeschiae* 9M and *P. odoriferum* Car 1 strains. MVs concentration of the samples was standardised based on the CE measurements of corrected peak areas. The leaves were placed in Petri plates or zip-lock plastic bags and incubated in a 90% humidity atmosphere at 28 °C for 48 h. After 24 and 48 h, the maceration zone was assessed visually. The macerated plant tissue was incubated at 28 °C for 48 h on the CVP agar plates. After incubation, the plates were assessed for the growth of pectinolytic bacteria.

### 4.15. MIC Evaluation

The presence of active β-lactamase enzymes was assessed in the MVs obtained from *P. odoriferum* Car 1 GFP-tagged strain, and a resistant to ampicillin *P. versatile* DPMP190 wild strain. The strains were grown in the presence of 200 mg L^−1^ ampicillin prior to vesicles isolation. Serial dilutions of ampicillin in 200 µL MH broth in two-fold decrements were prepared in the microtiter plate wells. The final concentrations of ampicillin in the wells ranged from 0 to 1.2 g L^−1^. *E. coli* DH5α bacterial suspension of 0.5 McF was prepared in MH broth and added to each well. Growth inhibition was assessed visually by turbidity changes in the wells, and by the resazurin assay. The assay was performed by the addition of 100 µL 0.02% resazurin to each well. The microtiter plate was subsequently incubated for 4 h. After that time, the colour change in each well was assessed.

### 4.16. Statistical Analyses

All of the experiments were done in triplicates unless otherwise noted. The data were analysed using a Student’s *t*-test or one-way Analysis of Variance (ANOVA) and Tukey’s multiple comparison test. Statistical analyses were performed in a commercially available software STATISTICA 13 (Tibco Software Inc., Palo Alto, CA, USA). All data are expressed as means ± standard errors of the means (SEMs). Differences were considered statistically significant at *p* < 0.05.

## Data Availability

The data that support the findings of this study are available from the corresponding authors (K.W.; M.W.), upon request.

## Supplementary Materials

The following are available online at https://www.mdpi.com/article/10.3390/ijms222212574/s1.
