# Supplementary material for: Membrane Vesicles of Pectobacterium as an Effective Protein Secretion System
Source: Int J Mol Sci. 2021 Nov 22;22(22):12574. doi: 10.3390/ijms222212574 (PMC8623790; doi:10.3390/ijms222212574)
Supplement: Supplementary file 1 [file ijms-22-12574-s001.zip › Jonca J corrected supplement.pdf]

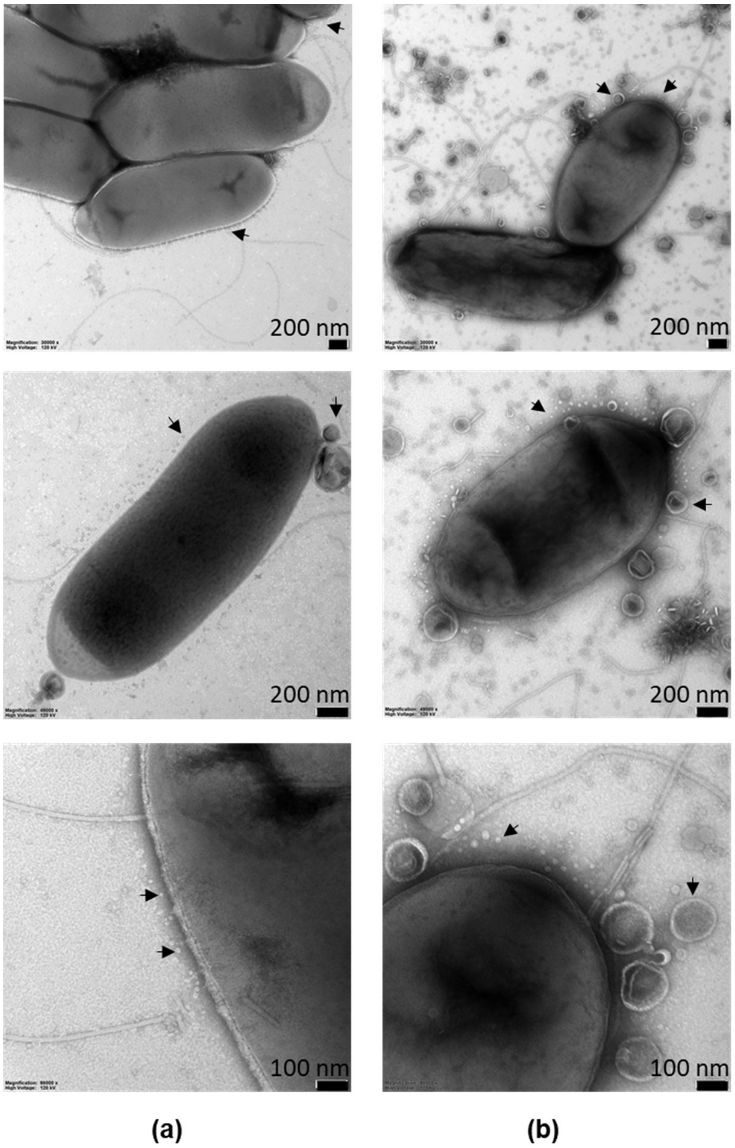

Figure S1. TEM images of *P. zantedeschiae* 9M bacteria grown on (a) the MH agar supplemented with 10% sucrose, and (b) the CVP agar plates. MVs are indicated by arrows. The size bar is 200 nm or 100 nm (bottom images).

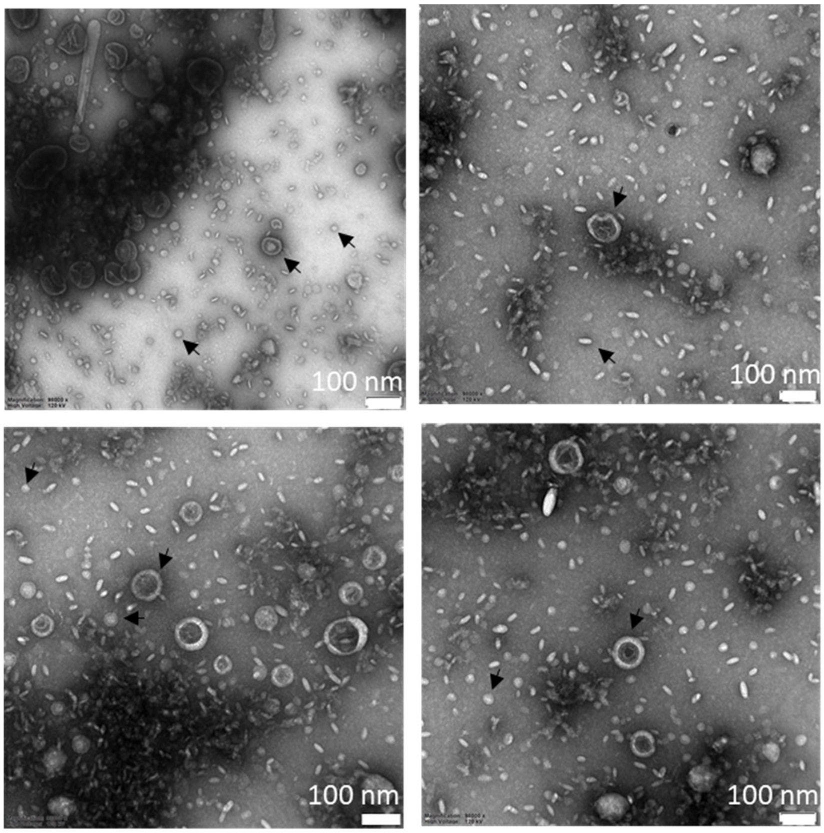

Figure S2. TEM images of the MVs produced by *P. zantedeschiae* 9M in the liquid M63 medium supplemented with 0.2% glycerol and 0.4% PGA. MVs are indicated by arrows. The size bar is 100 nm.

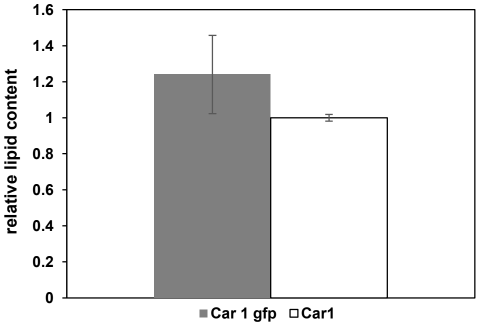

Figure S3. The relative lipid content of the MVs from the GFP-tagged *P. odoriferum* Car1 and the wild-type strain assessed by the DPH method. The obtained results were expressed as relative to the MVs derived from the wild-type strain. The data represent one of two reproducible experiments, expressed as mean ±SEM. Statistical significance was calculated using Student's t test ($P<0.05$).

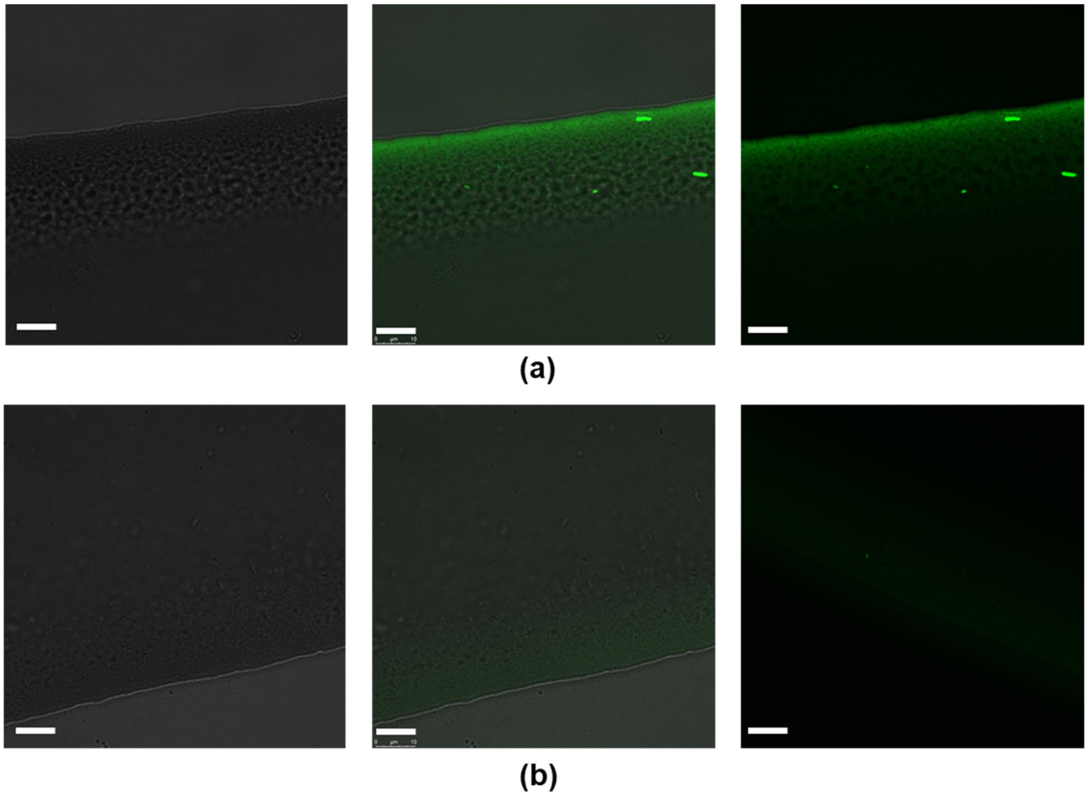

Figure S4. Confocal fluorescence microscopy images of the MVs from (a) the GFP-tagged *P. zantedeschiae* 9M and (b) the wild-type strain. From the left: transmitted light, merge, GFP fluorescence. The microscopic image is depicting a droplet of concentrated MVs on a microscopic slide. The MVs can be observed cumulating on the droplet edges, where the most intense fluorescence occurs. This effect does not occur for the wild-type strain, as depicted. The size bar is 10 μm.

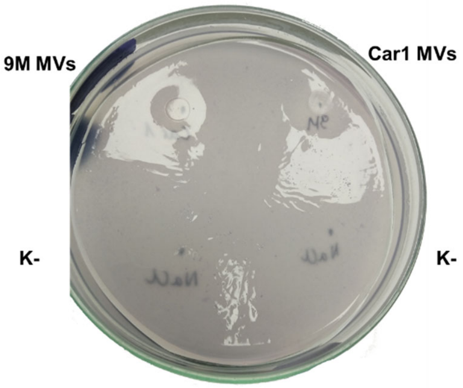

Figure S5. Representative results of the pectinase plate test on the CVP agar for the MVs derived from *P. zantedeschiae* 9M and *P. odoriferum* Car1. The formation of cavities indicates pectate degradation. Negative control systems contained PBS instead of the MVs suspension.

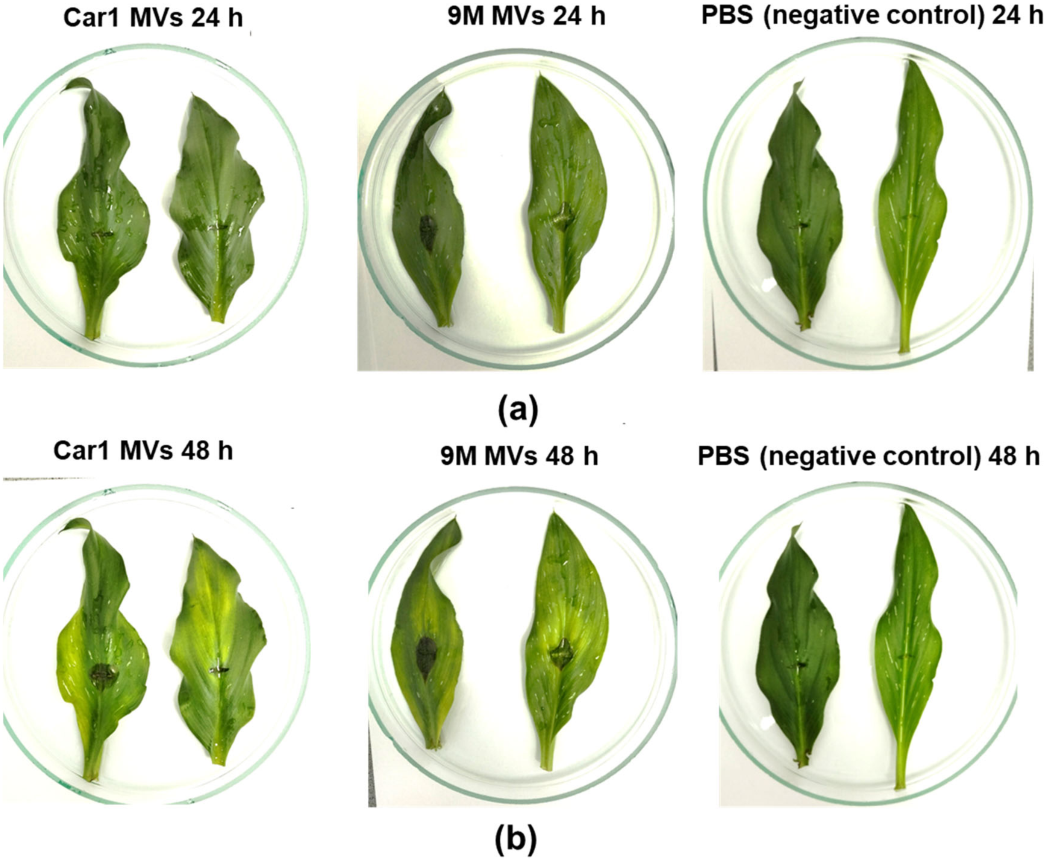

Figure S6. Representative images of the plant pathogenicity test on Calla lily leaves. Wounded lesions were made across the leaves with a pipette tip. The lesions were inoculated with the MVs derived from *P. zantedeschiae* 9M or *P. odoriferum* Car1. In the negative control, PBS was used instead of the MVs suspension. Maceration of the plant tissue was assessed after (a) 24 hours and (b) 48 hours of incubation.

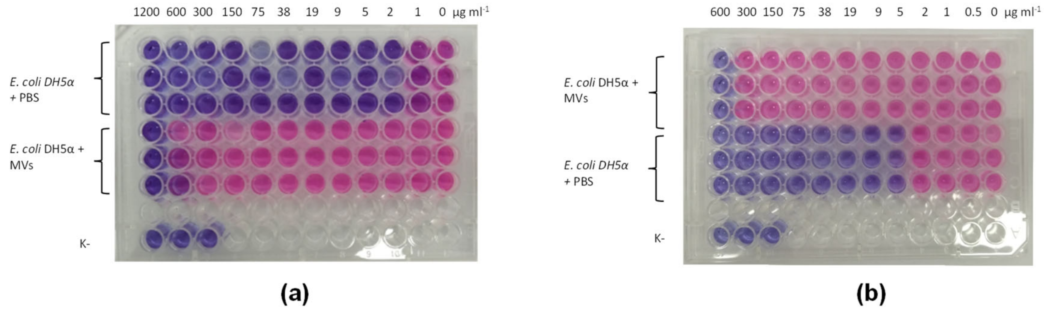

Figure S7. The MIC assay of ampicillin for *E. coli* DH5*α* in the presence of the MVs from ampicillin resistant (a) *P. odoriferum* Car 1 gfp and (b) *P. versatile* DPMP190 strains. The growth of ampicillin susceptible *E. coli* DH5*α* was compared in the MH medium containing the MVs or PBS, and increasing concentrations of ampicillin. The negative controls (K-) contained the MVs with no bacteria added. Ampicillin concentrations (µg ml^{-1}) are indicated above the microtiter plates images. The growth of bacteria was assessed with the resazurin assay. A pink colour indicates bacterial growth.

Table S1. The proteomic analysis of the MVs derived from the wild-type *P. zantedeschiae* 9M and the GFP-tagged strain. The wild-type strain was cultured in two different media, the M63 medium supplemented with 0.2% glycerol and 0.4% PGA and the M63 medium supplemented with 0.2% glycerol and 10% potato extract. The GFP-

tagged *P. zantedeschiae* 9M strain was grown in the M63 medium supplemented with 0.2% glycerol and 0.4% PGA.

UniProt entry name	Protein names	Gene name	# peptides 9M M63 0.2% glycerol 0.4% PGA	# peptides 9M gfp M63 0.2% glycerol 0.4% PGA	# peptides 9M M63 0.2% glycerol 10% potato extract
		**cytoplasmic proteins**			
A0A093RT18	Cysteine synthase (EC 2.5.1.47)	KP22_08815	20	13	4
A0A093RW32	Serine/threonine protein kinase	KP22_04685	32	0	0
A0A093SVG4	Sulfite reductase [NADPH] hemoprotein beta-component (SiR-HP) (SiRHP) (EC 1.8.1.2)	cysI	10	5	0
A0A093T2V1	Pyruvate dehydrogenase E1 component (EC 1.2.4.1)	aceE	40	28	1
A0A093UI17	Ribonuclease E (RNase E) (EC 3.1.26.12)	rne	19	9	0
A0A094U8G7	Chaperone protein ClpB	clpB	25	16	1
A0A0H3I270	Ferritin (EC 1.16.3.2)	W5S_2068	8	3	0
A0A0H3IF76	Ribokinase (RK) (EC 2.7.1.15)	rbsK	7	0	2
A0A0J5XQF8	Protein translocase subunit SecA	secA	17	14	0
A0A0J5XRY1	2-deoxy-D-gluconate 3-dehydrogenase	G033_11980	9	4	0
A0A0J5XTP8	Phosphoserine aminotransferase (EC 2.6.1.52) (Phosphohydroxythreonine aminotransferase) (PSAT)	serC	10	5	0
A0A0J5XVV4	Pyruvate kinase (EC 2.7.1.40)	G033_09265	16	10	0
A0A0J5XY19	Acetate kinase (EC 2.7.2.1) (Acetokinase)	ackA	12	3	0

A0A1D7YYA3	Threonine synthase (EC 4.2.3.1)	A7983_04370	11	5	0
A0A1D7Z3M3	Phosphoribosylaminoimidazole-succinocarboxamide synthase (EC 6.3.2.6) (SAICAR synthetase)	purC	12	3	0
A0A1D7Z400	Dihydrolipoyllysine-residue succinyltransferase component of 2-oxoglutarate dehydrogenase complex (EC 2.3.1.61) (2-oxoglutarate dehydrogenase complex component E2)	A7983_15280	10	6	1
A0A1D7Z4G6	DNA protection during starvation protein (EC 1.16.-.-)	dps	9	0	0
A0A1V2QZN7	Glutamate--tRNA ligase (EC 6.1.1.17) (Glutamyl-tRNA synthetase) (GluRS)	gltX	9	4	0
A0A221T5W1	Peptidyl-prolyl cis-trans isomerase (EC 5.2.1.8)	fklB	8	3	0
A0A221T6S2	Serine hydroxymethyltransferase (SHMT) (Serine methylase) (EC 2.1.2.1)	glyA	19	10	1
A0A221TEZ4	Elongation factor Tu (EF-Tu)	tufB	30	25	14
A0A2T3VSS6	Glutamate synthase large subunit	C9I36_19205	31	27	0
A0A2T3VSW9	Oligogalacturonate lyase	C9I36_18375	11	2	0
A0A2T3VT49	Isocitrate dehydrogenase [NADP] (EC 1.1.1.42)	C9I36_18440	23	5	0
A0A2T3VUP3	60 kDa chaperonin (GroEL protein) (Protein Cpn60)	groL	40	34	17
A0A3A4AJT7	Acetyltransferase component of pyruvate dehydrogenase complex (EC 2.3.1.12)	D5074_04595	18	18	2
A0A3S9XRA3	Tryptophan synthase beta chain (EC 4.2.1.20)	trpB	8	8	0
A0A3S9XXT4	Ribose-phosphate pyrophosphokinase (RPPK)	prs	14	9	1

	(EC 2.7.6.1) (5-phospho-D-ribosyl alpha-1-diphosphate) (Phosphoribosyl diphosphate synthase) (Phosphoribosyl pyrophosphate synthase) (P-Rib-PP synthase) (PRPP synthase) (PRPPase)				
A0A419AVX0	6,7-dimethyl-8-ribityllumazine synthase (DMRL synthase) (LS) (Lumazine synthase) (EC 2.5.1.78)	ribH	6	2	0
A0A419AWZ3	6-phosphogluconate dehydrogenase, decarboxylating (EC 1.1.1.44)	gndA	20	11	1
A0A426IYA4	Protein-export protein SecB	secB	5	3	0
A0A433NX46	10 kDa chaperonin (GroES protein) (Protein Cpn10)	groES	7	4	2
A0A4U7KAI5	Dihydroxy-acid dehydratase (DAD) (EC 4.2.1.9)	ilvD	19	7	0
A0A4U7KAN1	Elongation factor G (EF-G)	fusA	42	21	4
A0A4U7KBF9	Single-stranded DNA-binding protein (SSB)	EDI29_17775	4	2	0
A0A4U7KBS7	DNA-directed RNA polymerase subunit beta' (RNAP subunit beta') (EC 2.7.7.6) (RNA polymerase subunit beta') (Transcriptase subunit beta')	rpoC	45	41	1
A0A4U7KD30	Triosephosphate isomerase (TIM) (TPI) (EC 5.3.1.1) (Triose-phosphate isomerase)	tpiA	9	5	1
A0A4U7KGF4	Formate C-acetyltransferase (EC 2.3.1.54)	pflB	32	28	5
A0A4U7KI77	RNA-binding protein Hfq	hfq	6	7	0
A0A4U7KJB5	Elongation factor Ts (EF-Ts)	tsf	16	10	1
A0A4U7KL15	2,3-bisphosphoglycerate-dependent phosphoglycerate mutase (BPG-dependent PGAM) (PGAM)	gpmA	16	9	3

	(Phosphoglyceromutase) (dPGM) (EC 5.4.2.11)				
A0A4U7KM11	2,3,4,5-tetrahydropyridine-2,6-dicarboxylate N-succinyltransferase (EC 2.3.1.117) (Tetrahydrodipicolinate N-succinyltransferase) (THDP succinyltransferase) (THP succinyltransferase) (Tetrahydropicolinate succinylase)	dapD	5	4	0
A0A4V6ESF5	Uronate isomerase (EC 5.3.1.12) (Glucuronate isomerase) (Uronic isomerase)	uxaC	17	3	0
A0A4V6ESR5	Translation initiation factor IF-3	infC	6	2	0
A0A518VGF4	Transcription termination factor Rho (EC 3.6.4.-) (ATP-dependent helicase Rho)	rho	18	7	1
A0A518VJU5	Acyl carrier protein (ACP)	acpP	4	3	1
A0A518VKN8	2-dehydro-3-deoxyphosphooctonate aldolase (EC 2.5.1.55) (3-deoxy-D-manno-octulosonic acid 8-phosphate synthase) (KDO-8-phosphate synthase) (KDO 8-P synthase) (KDOPS) (Phospho-2-dehydro-3-deoxyoctonate aldolase)	kdsA	12	3	0
A0A518VMH5	Succinate--CoA ligase [ADP-forming] subunit beta (EC 6.2.1.5) (Succinyl-CoA synthetase subunit beta) (SCS-beta)	sucC	15	7	0
A0A518VNS1	ATP-dependent RNA helicase DeaD (EC 3.6.4.13) (Cold-shock DEAD box protein A)	deaD	12	3	0
A0A518VQA4	ATP synthase subunit alpha (EC 7.1.2.2) (ATP synthase F1	atpA	25	23	0

	sector subunit alpha) (F-ATPase subunit alpha)				
A0A6P1RRD2	Flavodoxin	fldA	4	1	0
A0A6P1RSR9	Aminotransferase (EC 2.6.1.-)	EO763_12775	17	1	1
A0A6P1RXT5	Glycerol-3-phosphate dehydrogenase (EC 1.1.5.3)	EO763_20725	22	5	1
A0A6P1RZH9	ATP synthase subunit beta (EC 7.1.2.2) (ATP synthase F1 sector subunit beta) (F-ATPase subunit beta)	atpD	24	17	1
A0A6P1RZJ5	Phosphoglycerate kinase (EC 2.7.2.3)	pgk	24	10	2
A0A6P1RZW4	UPF0325 protein GMX10_01590	GMX10_01590	8	0	1
A0A6P1SBS4	Tryptophan synthase alpha chain (EC 4.2.1.20)	trpA	6	0	0
C6D9B3	Chemotaxis protein CheW	PC1_2610	8	1	0
C6D9P1	PTS system, glucose subfamily, IIA subunit	PC1_0773	10	4	2
C6DCC9	Citrate synthase	PC1_1231	11	7	0
C6DCD7	Succinate--CoA ligase [ADP-forming] subunit alpha (EC 6.2.1.5) (Succinyl-CoA synthetase subunit alpha) (SCS-alpha)	sucD	9	1	0
C6DF33	2-oxoglutarate reductase (EC 1.1.1.399) (EC 1.1.1.95) (D-3-phosphoglycerate dehydrogenase)	PC1_3682	12	7	0
C6DF74	Histidine biosynthesis bifunctional protein HisB [Includes: Histidinol-phosphatase (EC 3.1.3.15); Imidazoleglycerol-phosphate dehydratase (IGPD) (EC 4.2.1.19)]	hisB	7	3	0
C6DFH6	Fructose-bisphosphate aldolase (FBP aldolase) (EC 4.1.2.13)	PC1_3700	10	8	5

C6DFS3	DNA-directed RNA polymerase subunit alpha (RNAP subunit alpha) (EC 2.7.7.6) (RNA polymerase subunit alpha) (Transcriptase subunit alpha)	rpoA	15	13	1
C6DJB6	Universal stress protein	PC1_4201	6	0	1
C6DJD5	Glutamine synthetase (EC 6.3.1.2)	PC1_4220	29	21	2
C6DKK2	Transcription termination/antitermination protein NusA	nusA	10	5	0
GFP	Green fluorescent protein	GFP	2	16	0
PURA	Adenylosuccinate synthetase (AMPSase) (AdSS) (EC 6.3.4.4) (IMP--aspartate ligase)	purA	14	3	1
Q6CZC6	Branched-chain-amino-acid aminotransferase (BCAT) (EC 2.6.1.42)	ilvE	12	1	0
Q6D011	Isocitrase (EC 4.1.3.1) (Isocitratase) (Isocitrate lyase)	aceA	10	0	0
Q6D088	Fructose-bisphosphate aldolase (FBP aldolase) (EC 4.1.2.13)	fbaA	13	9	4
Q6D0I8	Cell division protein FtsZ	ftsZ	12	3	0
Q6D3N0	S-(hydroxymethyl)glutathione dehydrogenase (EC 1.1.1.284)	adhC	8	5	0
Q6D4J9	Cold shock protein	ECA2391	6	3	2
Q6D4R2	DNA-binding protein	hns2	8	3	0
Q6D635	Phosphoenolpyruvate synthase (PEP synthase) (EC 2.7.9.2) (Pyruvate, water dikinase)	ppsA	14	2	0
Q6D7N1	Cold shock-like protein	cspE	5	3	3
Q6DA37	Aspartate ammonia-lyase (Aspartase) (EC 4.3.1.1)	aspA1	13	0	0

Q6DAE3	Diacetyl reductase [(S)-acetoin forming] (EC 1.1.1.304)	budC	7	0	2
Q6DAL5	DNA-binding protein HU-alpha	hupA	5	4	3
Q9WXA0	Tail core protein		2	6	0
A0A1M5PBI9	Chemotaxis protein CheA	SAMN05444147_105311	11	0	0
A0A6I7DBL4	3,4-dihydroxy-2-butanone 4-phosphate synthase	ribB	4	0	0
A0A6I7DFJ9	DNA-directed RNA polymerase subunit beta	rpoB	59	36	1
A0A6I7DH35	Phosphoenolpyruvate-protein phosphotransferase	ptsI	25	14	0
A0A6I7DQT6	Bacterioferritin	EH204_19515	6	3	1
A0A6I7DRV9	Aspartate-semialdehyde dehydrogenase	asd	10	9	1
		**ribosomal proteins**			
A0A249QAP7	50S ribosomal protein L2	rplB	14	13	1
A0A4U7KBP6	50S ribosomal protein L1	rplA	9	8	4
A0A4V6ERZ7	50S ribosomal protein L4	rplD	12	7	1
A0A518VGV9	30S ribosomal protein S7	rpsG	11	7	2
A0A518VGW1	50S ribosomal protein L22	rplV	6	7	1
A0A518VGW9	50S ribosomal protein L6	rplF	10	8	2
A0A518VGX4	50S ribosomal protein L16	rplP	7	5	0
A0A518VGX6	30S ribosomal protein S4	rpsD	4	10	1
A0A518VGX7	30S ribosomal protein S11	rpsK	7	5	0
A0A518VGY9	50S ribosomal protein L5	rplE	13	12	3
A0A518VGZ8	30S ribosomal protein S13	rpsM	9	4	0
A0A518VH32	30S ribosomal protein S3	rpsC	11	8	1
A0A518VH49	50S ribosomal protein L17	rplQ	6	8	1
A0A518VHP8	50S ribosomal protein L9	rplI	11	5	2
A0A518VHQ1	30S ribosomal protein S6	rpsF	6	6	1

A0A518VI99	30S ribosomal protein S16	rpsP	7	5	0
A0A518VLA4	30S ribosomal protein S1	EGD00_1281 5	32	24	2
A0A518VLM3	50S ribosomal protein L25	rplY	5	2	0
A0A518VN54	30S ribosomal protein S2	rpsB	13	12	1
A0A518VNT7	50S ribosomal protein L21	rplU	7	7	1
A0A518VPK1	50S ribosomal protein L13	rplM	6	5	1
A0A6P1S0U3	50S ribosomal protein L10	rplJ	8	7	3
A0A6P1S3Q1	50S ribosomal protein L3	rplC	6	5	0
C6DG57	30S ribosomal protein S5	rpsE	9	4	0
RS9	30S ribosomal protein S9	rpsI	9	8	2
A0A6I7DRM6	50S ribosomal protein L24	rplX	6	5	0
A0A518VPT6	50S ribosomal protein L7/L12	rplL	5	4	3
**cytoplasmic membrane proteins**					
A0A093T1T7	Sulfite reductase [NADPH] flavoprotein alpha-component (SiR-FP) (EC 1.8.1.2)	cysJ	10	13	0
A0A093UEQ3	Succinate dehydrogenase flavoprotein subunit (EC 1.3.5.1)	sdhA	12	2	0
A0A0H3I8G3	Cytochrome bd-I ubiquinol oxidase subunit I (Cytochrome d ubiquinol oxidase, subunit I)	W5S_3085	5	2	1
A0A0J5XNP0	Ribose import ATP-binding protein RbsA (EC 7.5.2.7)	rbsA	18	0	2
A0A6P1RV12	Enoyl-[acyl-carrier-protein] reductase [NADH] (EC 1.3.1.9)	fabI	8	7	0
C6DDF3	Lipoprotein	PC1_3344	12	10	0
C6DJD4	50S ribosomal subunit assembly factor BipA (EC 3.6.5.-) (GTP-binding protein BipA)	bipA	16	2	0
C6DJG8	ATP synthase subunit b (ATP synthase F(0) sector	atpF	7	6	3

	subunit b) (ATPase subunit I) (F-type ATPase subunit b) (F-ATPase subunit b)				
A0A1M5GMU1	Oligogalacturonide ABC transporter ATP-binding protein	SAMN05444147_101218	12	2	0
		**periplasmic proteins**			
A0A093RA42	Leucine ABC transporter substrate-binding protein	KP22_20180	14	1	4
A0A093T3Y9	Putrescine-binding periplasmic protein	KP22_03030	14	1	1
A0A0J5XMS3	Transaldolase (EC 2.2.1.2)	tal	15	8	1
A0A0J6AIS3	Histidine ABC transporter substrate-binding protein HisJ	G033_14170	8	1	0
A0A2T3VY27	Amino acid ABC transporter substrate-binding protein	C9I36_08780	8	8	1
A0A2T3VZ74	Sulfate transporter subunit	C9I36_07340	12	2	0
A0A426IUT6	Discoidin domain-containing protein	DMB83_003555	5	0	0
A0A4U7KL34	Thiol peroxidase (Tpx) (EC 1.11.1.24) (Peroxiredoxin tpx) (Prx) (Thioredoxin peroxidase) (Thioredoxin-dependent peroxiredoxin)	tpx	9	3	3
A0A4U7KMZ0	Amino acid ABC transporter substrate-binding protein	EDI29_01750	16	1	3
A0A6I6WW15	Ribose ABC transporter substrate-binding protein RbsB	rbsB	17	0	13
A0A6P1RNF8	Divisome-associated lipoprotein YraP	yraP	6	3	1
C6DAJ2	Chaperone protein Skp	PC1_0950	7	1	0
C6DEE1	Cationic amino acid ABC transporter, periplasmic binding protein	PC1_1584	8	0	0
C6DII0	Superoxide dismutase (EC 1.15.1.1)	PC1_4160	16	4	1

C6DJU7	Transport-associated protein	PC1_0454	6	0	0
Q6D987	Putative exported protein	ECA0732	6	0	0
Q6DAF0	Periplasmic serine endoprotease DegP-like (EC 3.4.21.107)	degQ	13	0	0
A0A6I7DG12	Tol-Pal system protein TolB	tolB	20	4	1
A0A6I7DN17	Peptidyl-prolyl cis-trans isomerase	EH204_19595	13	4	4
		**outer membrane proteins**			
A0A3A4ATX4	Outer membrane protein OmpW	ompW	9	6	10
A0A419B1E5	Porin	D5071_02990	6	5	5
A0A4U7KDA0	Toxin CdiA (Fragment)	EDI29_15430	2	0	10
A0A4U7KIX7	MipA/OmpV family protein	EDI29_02925	7	1	0
A0A6I6X068	Outer membrane channel protein TolC	tolC	11	5	0
A0A6P1RRG7	Porin	EO763_11905	5	1	0
C6DGP0	Filamentous hemagglutinin family outer membrane protein	PC1_0065	4	0	7
C6DJB5	Glutamate dehydrogenase	PC1_4200	11	14	0
C6DJX9	Toxin CdiA	PC1_2330	15	2	3
C6DK90	Major outer membrane lipoprotein Lpp	lpp	5	4	4
Q6D3I0	Outer membrane protein	ompX	9	5	3
Q6D7F1	Peptidoglycan-associated protein	pal	12	9	2
Q9RB08	Outer membrane lipoprotein Pcp	pcp	4	3	1
		**secreted proteins**			
A0A0J5XYH6	Flagellar hook-associated protein 1 (HAP1)	flgK	13	5	9
A0A0J6AP95	Flagellar hook-associated protein 2 (HAP2) (Flagellar cap protein)	G033_08615	11	7	12

A0A0M2EXB4	Hemagglutinin (Fragment)	KU74_20835	5	3	2
A0A1D7Z539	Endonuclease (EC 3.1.30.-)	A7983_16615	3	7	0
A0A1V2QYZ8	Flagellin (Fragment)	BSK71_20225	6	4	2
A0A433NE21	Uncharacterized protein	F126LOC_01 413	14	5	1
A0A6I6XCX0	Ig-like domain-containing protein	GT391_13040	24	5	2
C6DBM0	von Willebrand factor type A	PC1_3060	18	5	3
Q6D7Y9	F5/8 type C domain-containing protein	ECA1186	2	9	0
Q6D880	Endo-polygalacturonase (EC 3.2.1.15)	pehA	13	4	9
A0A1M5PFU6	Flagellin	SAMN05444 147_105355	19	12	8
A0A1M5PGH 7	Flagellin	SAMN05444 147_105354	18	11	9
A0A6I7D9X8	Flagellar hook protein FlgE	EH204_07515	7	0	0
		**unknown location**			
A0A0J5XRG5	Tail protein	G033_13365	12	20	0
A0A0J5XY41	Protein ElaB	G033_07880	4	0	0
A0A1V2R4S2	Dihydrolipoyl dehydrogenase (EC 1.8.1.4)	BSK71_10130	22	16	4
A0A4U7KF10	Malate dehydrogenase (EC 1.1.1.37)	mdh	18	4	5
A0A6I6X5W8	Carbamoyl-phosphate synthase large chain (EC 6.3.5.5) (Carbamoyl-phosphate synthetase ammonia chain)	carB	22	4	0
C6DF19	Bifunctional aspartokinase/homoserine dehydrogenase [Includes: Aspartokinase (EC 2.7.2.4); Homoserine dehydrogenase (EC 1.1.1.3)]	PC1_3668	13	17	0
C6DFZ7	SmpA/OmlA domain protein	PC1_1899	6	3	3

Q6D6E3	DUF3666 domain-containing protein	ECA1742	8	5	2
Q6D9H6	Putative membrane protein	ECA0639	4	3	1
A0A1M5MNT4	Uncharacterized protein	SAMN05444147_104139	0	4	0
